# Kleptomania Induced by Venlafaxine

**DOI:** 10.1155/2021/8470045

**Published:** 2021-09-10

**Authors:** Kumi Sakurada, Masashi Nibuya, Kazuo Yamada, Seishu Nakagawa, Eiji Suzuki

**Affiliations:** Division of Psychiatry, Tohoku Medical and Pharmaceutical University, Sendai City, Miyagi, Japan

## Abstract

*Introduction*. Kleptomania is an impulse-control disorder that results in an irresistible urge to steal. It is often observed as a comorbidity in patients undergoing pharmacological treatment for Parkinson's disease. Recurrent shopliftings are also observed in the clinical course of frontotemporal dementia. *Case Presentation*. After successful treatment of severe depression with venlafaxine at a dose of 225 mg/day, a 54-year-old euthymic female patient exhibited recurrent stealing behavior. After the diagnostic exclusion of frontotemporal dementia, kleptomania induced by venlafaxine administration was suspected. The symptoms of kleptomania disappeared with the gradual decrease in the venlafaxine dosage to 37.5 mg/day. *Discussion.* Venlafaxine is a dual serotonin-norepinephrine reuptake inhibitor. We considered two possible mechanisms to explain the pathophysiology of kleptomania in the present case: (1) increased dopaminergic neural transmission due to the inhibited dopamine reuptake by the norepinephrine transporter with a high dose of venlafaxine or (2) enhanced serotonergic neural transmission by the inhibition of serotonin reuptake by venlafaxine. In past studies, five cases of impulse-control disorder induced by selective serotonin reuptake inhibitors have been reported. This is the fourth report of venlafaxine-induced kleptomania and highlights the importance of considering the possibility of a rare side effect of kleptomania induced by antidepressant.

## 1. Introduction

Kleptomania has been categorized as an impulse-control disorder since the Diagnostic and Statistical Manual 3rd edition (DSM-III). An association has been indicated between kleptomania and affective spectrum disorders [1] and between kleptomania and obsessive-compulsive disorders [2]. Shoplifting and pathological gambling have often been indicated in patients with Parkinson's disease during prodopamine administration [3]. In addition, antisocial behaviors, including recurrent shoplifting, have also been observed in frontotemporal dementia (FTD) [4].

The present report discusses the emergence of symptoms of kleptomania in a 54-year-old female patient with depression being treated with venlafaxine. We considered two possible mechanisms associated with enhanced dopaminergic and serotonergic neural transmission after excluding possible comorbidity of FTD and a mood shift into a hypomanic or manic state from a depressive state.

## 2. Case Presentation

A 54-year-old female patient with increased anxiety and agitation was admitted to our psychiatric unit. She exhibited depressive mood, anhedonia, easy fatigability, headache, neck pain, appetite loss, and insomnia and insisted that she had no money to pay to the hospital. For the treatment, we gradually increased the dose of venlafaxine from 37.5 mg/day to 225 mg/day. After successful treatment of her major depression with venlafaxine at an oral dose of 225 mg/day for 10 weeks, the dose of venlafaxine had been maintained at 225 mg/day. She newly exhibited recurrent stealing behavior of food and electronic apparatus and destroyed drawings of other inpatients from the time point of 12 weeks after the administration of 225 mg/day venlafaxine. The stolen objects were not used and were discarded immediately. Maximum administration of 225 mg/day venlafaxine was terminated a month after the exacerbation of her stealing behavior. Repetitive shoplifting of items of little value to her was observed even after her discharge until the dosage of venlafaxine was decreased to 37.5 mg/day. The frequency of shopliftings was gradually decreased while the dose was titrated from 225 to 37.5 mg/day. The recurrence was not observed in the follow-up period of 6 months. She described the inability to resist the compulsion to steal useless objects and the stress release after the theft. Although increased appetite, especially for sweet food was observed concurrently after her recovery from depression, increased behavioral activity and sleep disturbance were not detected, and she was diagnosed with a euthymic mood. She had no history of physical diseases, had worked as a teacher in a public elementary school for 30 years, and had taken care of her parent for 10 years. She had no mood disorders before the present depressive episode, no comorbid eating disorders, obsessive-compulsive disorder, personality disorder, or antisocial conduct. She had been indicated as having a meticulous nature and a strong sense of responsibility for her premorbid personality. Although her father had been suffered from left hemiparesis for a decade due to the brain stroke, no family history of psychiatric diseases including major depressive disorder and bipolar affective disorder were not reported in her family. FTD was ruled out because there was no apparent brain atrophy on a magnetic resonance imaging examination and no cerebral hypoperfusion in the single-photon emission computerized tomography study using Iofetamine hydrochloride I-123. In addition, she obtained standard scores of 29/30 on the Mini-Mental State Examination and 18/18 in the Frontal Assessment Battery at bedside.

## 3. Discussion

This is the fourth report of venlafaxine-induced kleptomania presented by repetitive shoplifts [5–7]. Among the reported three patients, two received venlafaxine at high doses of 225–300 mg/day, and in the other report, the administered dose of venlafaxine was not indicated [5]. Venlafaxine is a serotonin-norepinephrine reuptake inhibitor (SNRI). Although the binding affinities of venlafaxine to dopamine transporter and dopamine receptors have been reported to be low, norepinephrine transporter (NET) has a high potential to reuptake dopamine. NET inhibition by venlafaxine potentiates dopaminergic neural transmission. The Ki value of dopamine to NET has been reported to be approximately 40–400 nM in comparison to the fact that the Ki value of norepinephrine to NET has been reported to be around 60–250 nM (PDSP Ki Database; https://pdsp.unc.edu/databases/kidb.php). Therefore, a high-dose venlafaxine treatment increases dopamine content in the synaptic cleft. The patient in the present study had received a high dose of venlafaxine when she exhibited recurrent shopliftings, and the kleptomania is likely due to increased dopaminergic activity induced by venlafaxine. Considering previous reports and the present case of kleptomania by venlafaxine, an increased high dose of venlafaxine is one of the possible risk factors to cause a rare side effect of kleptomania. In another report, duloxetine-induced kleptomania was reported, and the elevated dopaminergic tone was indicated to underly its pathogenesis [8]. While duloxetine also belongs to SNRIs, it is appropriate to conclude that duloxetine induces increased dopaminergic activity due to its high affinity (approximate Ki value of 230 nM; PDSP Ki Database) to the dopamine transporter.

Another possible pathophysiology is the enhanced serotonergic transmission by venlafaxine administration. Although early case reports have indicated the effectiveness of selective serotonin reuptake inhibitors (SSRIs) for the treatment of kleptomania [9, 10], a later double-blind study revealed that escitalopram, an antidepressant belonging to the SSRI family, had no effectiveness in the psychopharmacological treatment of kleptomania [11]. On the other hand, several studies reported the emergence of kleptomania by administration of SSRIs, including fluoxetine [12] and fluvoxamine [12, 13]. Escitalopram-induced pyromania, an impulse-control disorder, has also been reported [14]. Among the five cases of SSRI-induced impulse-control disorder, two cases were reported in children [13, 14]. The decreased tolerability of SSRIs in youth has been indicated by the increased occurrence of activation syndrome and jitteriness/anxiety syndrome accompanying with impulsivity, disinhibition, agitation, hostility, aggressiveness, and irritability [15–17]. The increased serotonergic transmission is another possible reason for exaggerating impulsive kleptomania in the present case.

A common feature of antidepressant-induced kleptomania is that it appeared newly and abruptly in a state of euthymia after the remission of depression during the period of antidepressant administration. The present case indicates the importance of considering the possibility of a rare side effect of kleptomania by antidepressant treatment and urges that physicians should promptly decrease the dose of antidepressants if such a side effect is observed.

## Data Availability

All data is included in the case report.
